# Enhancing SDN WISE with Slicing Over TSCH

**DOI:** 10.3390/s21041075

**Published:** 2021-02-04

**Authors:** Federico Orozco-Santos, Víctor Sempere-Payá, Teresa Albero-Albero, Javier Silvestre-Blanes

**Affiliations:** 1 Instituto Tecnológico de Informática (ITI), 46022 Valencia, Spain; forozco@iti.es (F.O.-S.); vsempere@dcom.upv.es (V.S.-P.); jsilves@disca.upv.es (J.S.-B.); 2 Departamento de Comunicaciones (DCOM), Universitat Politècnica de València (UPV), 46022 Valencia, Spain; 3 Departamento de informática de Sistemas y Computadores (DISCA), Universitat Politècnica de València (UPV), 46022 Valencia, Spain

**Keywords:** IWSN, SDN, TSCH, QoS, DetNet, slicing

## Abstract

IWSNs (Industrial Wireless Sensor Networks) have become the next step in the evolution of WSN (Wireless Sensor Networks) due to the nature and demands of modern industry. With this type of network, flexible and scalable architectures can be created that simultaneously support traffic sources with different characteristics. Due to the great diversity of application scenarios, there is a need to implement additional capabilities that can guarantee an adequate level of reliability and that can adapt to the dynamic behavior of the applications in use. The use of SDNs (Software Defined Networks) extends the possibilities of control over the network and enables its deployment at an industrial level. The signaling traffic exchanged between nodes and controller is heavy and must occupy the same channel as the data traffic. This difficulty can be overcome with the segmentation of the traffic into flows, and correct scheduling at the MAC (Medium Access Control) level, known as slices. This article proposes the integration in the SDN controller of a traffic manager, a routing process in charge of assigning different routes according to the different flows, as well as the introduction of the Time Slotted Channel Hopping (TSCH) Scheduler. In addition, the TSCH (Time Slotted Channel Hopping) is incorporated in the SDN-WISE framework (Software Defined Networking solution for Wireless Sensor Networks), and this protocol has been modified to send the TSCH schedule. These elements are jointly responsible for scheduling and segmenting the traffic that will be sent to the nodes through a single packet from the controller and its performance has been evaluated through simulation and a testbed. The results obtained show how flexibility, adaptability, and determinism increase thanks to the joint use of the routing process and the TSCH Scheduler, which makes it possible to create a slicing by flows, which have different quality of service requirements. This in turn helps guarantee their QoS characteristics, increase the PDR (Packet Delivery Ratio) for the flow with the highest priority, maintain the DMR (Deadline Miss Ratio), and increase the network lifetime.

## 1. Introduction

The application of the concept IoT (Internet of Things) to the field of industrial automation has served to develop the concept of Industry 4.0 [1]. Thanks to the IoT it is possible to collect and process information in the place where it is generated, placing devices with the capacity to execute local actions or remote orders based on this information [2]. All this has been achieved at a relatively low cost and is easily replaceable and not very intrusive. However, time requirements, reliability, and security are more stringent in industrial environments than in the IoT. That is why, within the IoT, there is a branch for industrial use that consists of more robust devices and protocols, which must adapt to the needs of industry, and this is known as the IIoT (Industrial Internet of Things).

The IWSN is an important part of the IIoT [3]. These networks are composed of multiple nodes, usually low-power and low-cost devices, specifically oriented to collection and delivery of the parameters of interest to a central node, thus ensuring QoS [4]. In addition, due to their size and battery operation, they are devices that generally have processing and memory limitations, although operating wirelessly allows them to be very flexible, easy to replace, and adaptable to hostile environments [5]. These devices have been successful in industry and have been the driving force behind the revolution in industrial processes. However, today’s convergence processes require greater control over communication infrastructure, which must have a high degree of flexibility and reconfiguration capability. In modern industrial processes, information flows are often completely deterministic and guaranteed. It is for this reason that IWSNs must evolve towards new network paradigms such as Software Defined Networks (SDN) [6]. These allow problems that are intrinsic to WSNs, such as the difficulty of administration and the capacity for dynamic reconfiguration, to be solved [7,8].

Although Industrial Wireless Sensor Networks (IWSNs) have been refined with protocols such as RPL (Routing Protocol for Low-Power and Lossy Networks) and Time Slotted Channel Hopping (TSCH) [9], which allow scheduling of transmissions to avoid collisions and interference [10], the ability to reconfigure the network is limited. In addition, the flexibility and determinism of the network is affected by the autonomous decisions of each node. However, the use of a centralized approach with full knowledge of the network topology allows for optimized routing, TSCH scheduling, and reduced processing load on the nodes. However, the main drawback of centralized approaches continues to be that they involve adding an additional signaling traffic flow for control information. This reduces network performance as has been shown in previous research work [11,12,13]. The main drawback to these research works is that they do not represent a significant change in the operation of the nodes. That is why a more disruptive approach is needed, such as SDNs, where the network is divided into two different planes, control and data, and the nodes operate according to the controller’s instructions. This simplifies the administration of the network, because applications are generated oriented to the controller, which is responsible for modifying the behavior of the nodes according to the particular needs of each application. Software Defined Networking solution for Wireless Sensor Networks (SDN-WISE) [14] is a very flexible deployment that allows a total configuration of the controller on the nodes and that is currently under development.

So far, current studies using SDN-WISE have focused on specific aspects, such as “Priorities” [15], where from the level of congestion in each node, the probability of discarding the packets is modified and the controller modifies the routes. The routing process [16] is where traffic is prioritized according to its sensitivity to delay and packet loss; and uses a multipath routing protocol, with a metric composed of congestion, battery level, and RSSI (Received Signal Strength Indicator) values of the nodes. Mobility and TSCH are dealt with in [17], where it is demonstrated that, from high update levels, the controller can adapt to changes produced by a mobile node. Most of these studies are performed on CSMA and only the study shown in [17] deals with TSCH, although because the goal was mobility, details of TSCH such as the scheduler and the amount of traffic control were not addressed.

SDN-WISE is a protocol on which multiple relevant element studies have been conducted in IWSNs. However, there is a gap in the integration of each of these elements, which are currently necessary for the DetNet (Deterministic Networks). For this reason, starting from the open version of SDN-WISE, the following are proposed:

- Establish a traffic manager in the controller, which receives the expected network flow descriptors, deadline, delay sensitivity, and packet loss as parameters.
- Incorporate a Routing process that generates multiple routes for the traffic manager’s flows.
- Incorporate a TSCH scheduler that receives the routes and plans the flows according to their deadlines.
- Add to the SDN-WISE the 802.15.4e TSCH MAC layer on Contiki NG, to support the new generation nodes.
- Modify the original elements of the SDN-WISE such as the OpenPath, to send information on the TSCH scheduling and avoid an increase in control traffic.

The joint use of these elements was evaluated by simulation and with a testbed. The parameters to be evaluated were the amount of control traffic, delay, packet loss, DMR, and network lifetime.

The rest of the paper is organized as follows. Section 2 presents the related work. Section 3 proposes the working architecture and the deployment to be carried out, where the application plane protocols and the modifications made to the SDN-WISE protocol to obtain the SDN TSCH network are examined more deeply. Section 4 describes the scenario and the tests to be carried out both in the simulation and in the testbed. The results obtained are shown in Section 5. Finally, the conclusions of this article are presented in Section 6.

## 2. Related Work

### 2.1. SDN

The SDN paradigm involves managing networks by separating the Control Plane from the Data Plane, where centralized equipment known as a controller is in charge of defining the forwarding rules based on a total topological knowledge of the network (Data Plane) and the policies established for each of the flows. The nodes receive these rules and store them as flow tables. After this, they simply have to search within this table and execute the actions sent by the controller (Control Plane). This approach makes the network programmable since the controller can modify the flow tables of each sensor [7]. In addition, it allows the traffic to be divided into flows, which is much more complex than segmentation by IP or MAC, due to the fact that multiple variables are analyzed at a local and global level. Thus, it is possible to significantly improve network performance, increase QoS, and prevent possible bottlenecks.

The advantages of SDN over the traditional approach are essentially versatility, flexibility, and manageability. This is because the nodes are no longer dependent on applications but are adaptable [7], which allows its operation to be modelled at any time through the commands of the controller. The applications in SDN are oriented to the controller, where the information flows and the behavior of the nodes are managed. The greatest efficiency of SDN is achieved in the long term where its evolution and adaptability capabilities are needed. For example, it is possible to solve the incompatibility of protocols such as Zigbee, 6TiSCH, and 6LoWPAN, as they share the lower layers (802.15.4) and the upper layers are defined by software and can be modified “on the fly” [6].

Thanks to the capabilities offered by the SDN, especially the flexibility, it is possible to generate any type of controller-oriented application, and new possibilities are opened up to address the wide range of challenges presented by WSNs. Improvements can be added with the implementation of an SDN such as energy efficiency, routing, mobility, security, reliability, and the capacity to adapt when a node fails and QoS [18]. QoS is becoming more and more important, because in the new-generation networks, there are traffic flows from many different sources. Therefore, each type of traffic must be guaranteed different QoS parameters [19]. In industrial networks, it is vital that they offer predictable behavior; for this reason, the times for sending and receiving information must be guaranteed and synchronized, making the network highly deterministic. In order to achieve this, multiple factors must be taken into account in the topology (congestion, link quality, etc.), as well as the segmentation in flows by type of traffic and integration with other types of networks. Therefore, the SDN approach is more suitable to achieve this goal as it has the ability to recognize, monitor, and configure each of these stages

### 2.2. TSCH

To achieve a better adaptation of the WSN within the industrial sector, the 802.15.4e standard includes different MAC protocols such as LLND, DSME, and TSCH, in order to adapt to different applications, reduce power consumption, increase transmission reliability, and achieve greater determinism in the network by eliminating much of the randomness of CSMA. According to [20], TSCH has better performance in terms of latency and energy consumption. In addition, it increases reliability in aggressive scenarios, where frequency hops allow interference to be mitigated.

TSCH allows scheduling of transmission and reception to avoid collisions and interference. To do this, it divides the time into a fixed number of timeslots that are grouped into slot frames, which are cyclical. The most common duration of each timeslot is 10 ms, enough time to be able to transmit a frame of the maximum size (127 Bytes), wait for the ACK (Acknowledgement) from the receiver, and process the packet. The frequency division is done with the assignment of a channel offset, which is used to determine the physical channel, therefore a timeslot and a channel offset must be defined for each transmission. The combination of these two parameters is a cell. Cells can be shared or dedicated. In the case of Shared, the CSMA-CA (Carrier Sense Multiple Access with Collision Avoidance) scheme is followed where the medium is monitored before transmissions to reduce collisions. On the other hand, if it is Dedicated, there are strong time restrictions, since a unidirectional link is established between two nodes and only the source node can transmit during the timeslot and in the specific offset channel. The scheduler allows the operator to establish what action the node is going to do in each timeslot. The node will be able to receive, transmit, or turn off the radio. For this to work correctly, the nodes covered by the scheduler must be correctly synchronized so that the transmission and reception timeslots coincide. This is achieved with the EB (Enhanced Beacon) packet exchange, which includes information about the ASN (Absolute Sequence Number) that the nodes take as a time reference and determines the current timeslot. The ASN information also allows the clock drifts to be compensated. ASN and Channel Offset are used in Equation (1), to determine the physical channel to be used. This generates a rotation of the physical channels in each slot frame that helps reduce the points of interference and path fading, increasing reliability. The problem here is that a large number of channels have a longer synchronization time [21].

In a slot frame, the columns represent the timeslots, and the rows represent the Channel Offset, which helps to determine which Transmission and Reception channel each node should use. It is an offset and not a fixed channel, because the total group of physical channels has a rotation according to Equation (1).

(1) $$\mathrm{frequency}=F[(\mathrm{ASN}+\mathrm{ChannelOffset})\mathrm{mod}N_{c}]$$

In the 2.4 GHz band, it is possible to have up to 16 channels available. The number timeslots determine the size of the slot frame. This parameter has implications for the throughput, latency, and convergence time, so its size should be chosen based on the applications. To ensure that timeslots rotate through all channels, the size should be a prime number [22].

There are several types of TSCH schedulers; centralized such as TASA (Traffic Aware Scheduling Algorithm) [11,12] and AMUS (Adaptive Multihop Scheduling) [13], and others that are distributed such as DeTAS (Decentralized Traffic Aware Scheduling) [23], Wave [24], and Orchestra [25], as well as some variants of these such as Escalator [26] and ALICE (Autonomous Link-based Cell scheduling) [27]. The tests shown in [13] indicate that AMUS performs better than TASA regarding reliability and latency. Although it is designed for a static network, it does not take into consideration the impact of signaling traffic on data traffic. It allocates additional slots for cases where retransmission is needed but does so on the same route. The problem with centralized processes is the signaling traffic they generate. However, these are not focused on SDN, where an increase in signaling traffic is assumed in exchange for flexibility and reconfiguration capacity.

Orchestra is a widely used protocol in TSCH networks due to its simplicity and high performance, especially in PDR. Because of this and the large number of studies that have used it, it is implemented natively in Contiki NG. Orchestra is not considered strictly distributed, as it does not require additional exchange of information by the nodes, but neither is it really a stand-alone protocol, as there is an exchange of control packets, although they are not exclusive. Orchestra reuses the control traffic of the RPL protocol, to build up the scheduling autonomously. Control traffic and speed of response is the major drawback of centralized schedulers, because it generates problems in data traffic.

### 2.3. SDN-WISE

SDN-WISE is a protocol that extends the SDN paradigm to WSNs. It is based on the OpenFlow protocol, a protocol designed for wired networks. This has involved making the necessary adaptations, as there are many challenges arise when it is deployed in wireless environments [7]. To meet these challenges, a profound change in the OpenFlow protocol was required, such as being able to make decisions according to local states without controller intervention (Statefull) or being able to perform packet aggregation to reduce congestion. Due to the complexity of the changes, protocols have been created that allow OpenFlow concepts to operate within the restrictions of wireless environments such as SDN-WISE. These changes are aimed at reducing control traffic and conserving network bandwidth, since in WSNs, there is no physical isolation between the two flows, so control traffic has a direct impact on network performance. However, the advantages of an SDN deployment, such as versatility, flexibility, and being easy to manage [28], are key elements within the WSN, because the traffic flows in the network can be programmed and modified by the controller, simply by modifying the flow table in each node.

**Figure 1:**
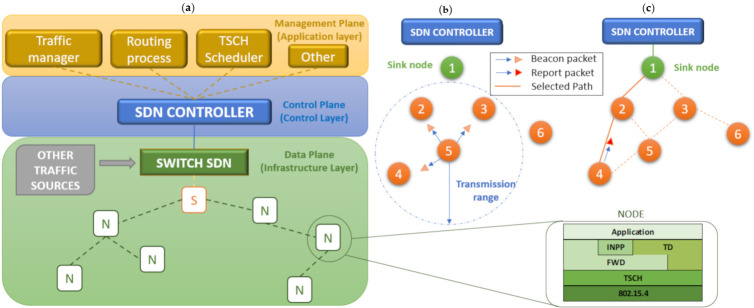
(**a**) Software Defined Network (SDN) architecture, (**b**) beacon packet, (**c**) report packet.

The SDN-WISE framework is mainly composed of five layers: Application, INNP (In-Network Packet Processing), TD (Topology Discovery), FWD (Forwarding), and the physical layer that will be 802.15.4e and TSCH-MAC layer [14]; the last two are standardized. The three layers where the operation of SDN-WISE is concentrated are described below, in Figure 1a.

Topology Discovery: his layer is responsible for discovering the adjacent nodes and informing the controller of its neighbors. To discover the adjacent nodes, all the nodes generate beacon packets, which are periodically sent in broadcast mode to inform all the nodes in the coverage range. Nodes that receive this packet add this node to their neighbor table. This information of all the adjacencies of each node is sent to the controller in a Report Packet. The controller receives the Report Packets from each of the nodes and builds a topology based on the information received, obtaining a global view of the network.

In-Network Packet Processing. Is a layer used for data aggregation. Small packets can be combined when they have the same destination to minimize network congestion. It should be noted that the maximum size of an SDN-WISE packet is 116 Bytes, and this limit cannot be exceeded.

Forwarding. Processes outgoing packets according to the WISE flow table. This table is composed of the flow rules sent from the controller that have three sections: matching rule, action, and statistics. The matching rules contain the fields that must be compared in the packets and the expected value. The packet can be inspected in any of its bytes as opposed to OpenFlow, which only compares values of the header. It is possible to add up to three matching rules for each flow rule. When the packet passes through the rules successfully, the action is executed, which can be of five types: Drop, Forward, Sleep, Modify, or INNP, and is counted in the statistics.

The forward action is executed according to the ID node, and this identifier is 2 bytes long. The use of these short addresses is common in computers that have memory and packet size limitations. In this way, the amount of memory and useful information transmitted is optimized. The nodes can be configured manually or with the two least significant bytes of the MAC address. In this work, the 2 bytes represent the number of the network node.

SDN-WISE sends different types of packets that allow the node to communicate with the controller. All packets in SDN-WISE have a 10 Byte Header consisting of the following fields: Packet Length (1 Byte), Network ID (1 Bytes), Source Node (2 Bytes), Destination Node (2 Bytes), Packet Type (1 Byte), TTL (Time To Live) (1 Byte), and Next Hop (2 Bytes). From this point on, the information included depends on the type of packet, as described below.

Data (Type 0). This type of packet is responsible for sending common traffic, such as sensor measurements. The bytes after the header make up the payload, and the following priorities are applied to this type of packet.

Beacon (Type 1). It is a Broadcast message that is sent to inform the nodes that are within range. It includes information about the battery status (0xFF = Full, 0 × 00 = Empty) and the distance to the sink (number of hops). More parameters can be added, for example, the level of congestion measured by the buffer occupation. An example of the sending of this packet is shown in Figure 1b. Node 5 sends the beacon packet (broadcast) to all those nodes that are within its transmission range. In the example, nodes 2, 3, and 4 would receive this packet.

Report (Type 2). This type of packet is used by the controller to create the network topology. By default, each node sends this packet periodically every 2× Beacon periods, with 5 s the default value for the beacon period. The packet includes the number of neighbors and information about each one, such as the ID node and the RSSI. In Figure 1c, node 4 sends a report packet to the controller to indicate its neighbors and does so by selecting the shortest route, which it knows because the beacons contain the distance to the sink.

Request (Type 3). When a packet does not have a match in the flow table, it is sent to the controller to ask what it should do with this type of traffic. The controller responds with a Type 4 message, with the flow rules.

Response (Type 4). The controller sends type 4 messages with flow rules when a node makes a request for traffic that is not specified in its flow table.

**Figure 2:**
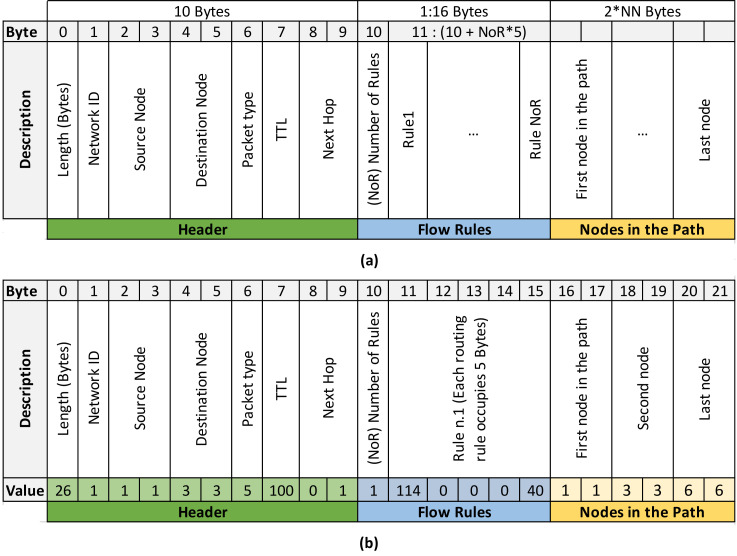
Structure of the original OpenPath packet. (**a**) Generic example, (**b**) example with three-node Route and one Flow Rule.

OpenPath (Type 5). This packet takes care of installing a specific flow rule on multiple nodes with a single transmission from the controller. The nodes where they are installed are part of a route. The packet is received by one node, processed, and then sent to the next node in the route. This process reduces control traffic. In Figure 2a, an OpenPath packet as defined in the SDN-WISE protocol is displayed [14]. The bytes after the header are used to define the Flow Rules and the nodes in the route as explained below. Byte#10 indicates the number of matching rules that the node should install, up to a maximum of three. Each matching rule has a set length of 5 bytes and is composed of a condition and an action. In the condition, you can compare specific values of the packet in any of its fields, including payload. When this condition is met, the action is executed. The next group of bytes are those related to the Nodes in the Route. Generically, this group will start at Byte#(10 + NoR × 5 +1), since it depends on the Number of Rules (NoR). In these bytes, in order of forwarding, the identifier of each of the nodes that make up the route is included. Each node is identified with two bytes as already indicated. When a node receives an OpenPath packet, the Flow Rules are sought among the group of nodes and are installed taking into account the following node in the group of nodes. When it finishes processing the packet it forwards it to the next node in the node group. This process is repeated in each node until it reaches the final destination, installing the Flow Rules in all the nodes of the route with a single packet.

Figure 2b also shows the OpenPath packet structure, but in this case, for a specific example: The path is composed of three nodes, namely nodes 1-3-6 and 2 hops (1🡢2, 2🡢3). A single routing rule (NoR = 1) has been defined, so in this case, of the 16 possible bytes to be occupied by the “Flow Rules”, only 6 bytes have been occupied. In the case of the example, from Byte#16, the nodes involved in the route are identified, using two bytes to identify each of them.

Config (Type 6). Used to set configuration options on the node, such as beacon period, report, or include Flow Rules specific to a node.

RegProxy (Type 7). Each sink node sends this packet to the controller to announce its existence, in this it sends the sink’s identifier and its MAC. With this information the controller decides whether to accept communication with this sink. After communication is established control packets of the whole WSN pass through this link.

### 2.4. Background and Motivation

Despite the advantages of SDN, its application towards WSN environments is not direct because SDN is a paradigm initially created for data center equipment, which has multiple simultaneous links with bandwidths of tens of Gb. On the other hand, WSN equipment is very limited in bandwidth and in the number of simultaneous links. For this reason, a range of research work has been carried out on the implementation of SDN in WSN, based on Tie Luo’s ideas in [7], where they try to move the OpenFlow protocol, the standard for wired networks. However, due to the great differences in the WSN environment, the amount of modifications that had to be made to the protocol made it unfeasible. From here, different SDN frameworks applied to sensor networks have emerged. TinySDN [29] is based on the TinyOS operating system and its application to SDN is limited due to the only two actions (Drop and Forward) performed by the flow table. SD-WSN [6] contains all the features present in the OpenFlow sensor and extends it through functions such as “in-network data aggregation”, which allows flows to join that share routing characteristics (INNP). uSDN [30], is a framework that is optimized to reduce the control traffic of the SDN infrastructure. It uses the 6TiSCH protocol and its new elements to create TSCH scheduler in a hybrid way (Centralized and Distributed). With this scheduling, a slicing of the network is performed to send the signaling traffic. However, the use of standardized top layers reduces the flexibility of the software-defined network. Atomic SDN [31] transmits the signaling traffic in a more efficient, fast, and isolated way from the other processes, using synchronous flooding mechanisms but due to the different types of flows it becomes complex, since it needs an algorithm for each type. A lot of research in SD-WSN has been focused on routing optimization and data flow control, but other aspects such as topology control can be improved, as it has been done in [32]. In [28], SD-WISE is proposed, an SDN proposal for WSN that improves the definition of flow rules, where it is possible to analyze any byte of the package unlike the previous frameworks that were limited to analyzing specific fields as in wired networks. SD-WISE focuses on flexibility and security. It considers duty cycles for energy optimization and transmission power control through actions, enables NFV (Network Function Virtualization), and considers security aspects (trusted). SD-WISE is stateful, it keeps the state information inside each sensor and that state can be modified through the execution of actions. The definition of the Rules is more flexible. An evolution of this protocol is SDN-WISE [14], which has been used in recent work such as [17] and that will also be the protocol used in this paper. This protocol performs multiple actions on the flow table (Drop, Forward, Sleep, Modify, INNP), and has incorporated elements of minimal TSCH but does not yet take into account scheduling from the controller. It can be seen how this framework has evolved and changed by the contributions in different studies. For example, in [15], where a buffer status indicator is added to inform the controller of the congestion levels of each node and balance the traffic. In addition, in [33] the response of the SDN approach with moving nodes is analyzed.

The general drawback of these frameworks is the lack of integration between the processes since they focus on the elements of routing and TSCH scheduler separately. A complete Software defined WSN deployment must have a total centralization of the network, where the routing processes, TSCH scheduler, and traffic foreseen in the network are related. In addition, subsequent integration with the wired SDN must be scheduled in order to reserve the necessary resources, although this integration is not investigated in this article.

## 3. SDN over TSCH

Figure 1a shows the SDN architecture where the two main planes of any SDN are distinguished, Control and Data. In addition, a layer is included on the Control Plane, the Application layer, or Management Plane, where different services are offered through different SDN applications (SDN App). These applications are oriented directly to the controller. The Traffic manager, the Routing process, and the TSCH Scheduler in the Application Layer are contributions made in this paper. This will allow segmentation and application of different qualities of service to each of the flows, thanks to the use of different routes (Routing) and assignment of the cells in the TSCH scheduler (deadline, retransmissions). In the Control Plane, the SDN controller that is responsible for governing and directing the way data are transported is located. Finally, in the lower level of the architecture, the Data Plane is located, and here are the SDN devices, such as the switch, which is in charge of transporting the data based on the instructions received by the controller. Furthermore, in this third layer, a WSN is deployed, placing in first place the Sink (S), which is linked to the switch and the rest of the Nodes (N), which are fixed. The traffic from the SD-WSN is integrated into the wired SDN network through the SDN switch. This is an additional flow that must be managed. However, this configuration is beyond the scope of this paper. Therefore, in this paper, the switch will be completely transparent.

The use of SDN in the WSN implies a total knowledge of the network in exchange for increasing control traffic and convergence time. This extra traffic is due to the periodic messages from the nodes, which give information about the neighbors, the battery, RSSI, and the level of congestion.

With a complete topological knowledge of the network, TSCH allocation can be done in an optimized way based on various parameters, such as periodic traffic, level of congestion, separation between nodes, and routes with different QoS policies. This assignment can be adapted according to the node congestion and link quality statistics. The aim is to achieve different routes on which the different traffic flows can be distributed, guaranteeing the quality-of-service parameters. The use of multiple routes allows traffic to be distributed over as many nodes as possible, achieving a load balance, flexibility, and fast adaptability to network changes. Additionally, congestion and energy consumption of the central nodes are reduced.

With this in mind, the following sections show the integration of the different components until we have a fully programmable sensor network, from the application plane in the controller to the medium access control in the nodes. The first stage is the integration of the three processes of the application layer: QoS, Routing Algorithm, and TSCH Scheduler; the second stage is the control of the MAC layer in the nodes, which includes the addition of the TSCH layer to the SDN WISE, and the definition of the packet that allows the configuration of the MAC layer with the result of the previous stage.

### 3.1. Traffic Manager and QoS

Because this implementation of SDN-WISE allows control over the MAC layer (TSCH) and the Forwarding layer of the WSN, it is possible to apply DetNet concepts [34]. These are based on the requirements of each flow and ensuring the QoS parameters, by means of resource reservation. This information per flow is contained in the controller itself in the traffic manager.

The flow descriptor in the DetNet allows the traffic specification parameters such as the packet size, transmission time interval, and the maximum number of packets per interval [34] to be set. The network uses traffic specification to allocate resources on the network. In our case, the flow descriptor is formed by the fields: Priority, deadline [ms], source node, and destination node. Each of the entries in the traffic manager is considered a flow descriptor. The QoS requirements are related to the priority, as can be seen in Table 1. The controller organizes the flows in this table by priority and deadline because the order is important in the subsequent processes.

Generally, QoS relies on traffic classification and tagging to allocate network resources according to the different classifications made. An optimal network design should not neglect QoS requirements, as this could lead to poor communications. However, implementing QoS techniques in WSNs is not so easy since this type of network is designed for low-data-rate applications and not for real-time applications [35]. This is why SDN-based WSNs can offer optimized QoS, as controller-oriented programmable applications can be developed to implement and manage QoS requirements related to sensor nodes [36].

In order to establish the real time requirements for the different types of data, three levels of priority will be established; see Table 1. Priorities are set according to time delay sensitive and packet loss rate sensitive data. In the case of priority 1, the highest priority level, the data are delay sensitive and loss-rate sensitive. That is to say, these are the routes with the least congestion and the least number of hops on the route. The typical traffic type for this priority is events. Priority 2 has been established as packet-loss sensitive, routes with the best link quality and low levels of congestion. In this case, it is the transmission of information that must reach its destination without any packet being lost, even if it arrives later. Finally, priority level 3 is set for periodic data such as telemetry measurements. In this case, the data are not sensitive to time delay and packet loss rate. However, periodic data can be assigned to any priority. This depends on the specific application that uses these data.

Based on these priorities, delay and packet loss metrics must be retained for each of the three types of traffic. In addition to this, in the case of detecting the failure of one of the nodes of the route, the flows with higher priority will be sent using the resources of the lower priorities until the controller reconfigures the routes. This flexibility will reduce the impact of the failure on the higher priority traffic.

### 3.2. Routing Process

Routing is the central phase of the integration process in SDN-WISE. It will be in charge of positioning within the WSN the flows defined in the traffic manager. This process is carried out by selecting the hops from origin to destination, forming a route. Route selection has a critical impact on network performance. For this reason, there are many studies that propose routing protocols to obtain the best performance in certain situations such as reducing energy consumption, increasing throughput, or implementing QoS [37]. One of the most common currently among WSNs is the RPL where it is possible to configure OF (Objective Functions) with multiple parameters. However, multiples OF may obtain routes that converge on the same nodes, and this is reflected in congestion at the central nodes [38].

In this phase, segmentation of the traffic flows by multiple routes is proposed, so a multiroute routing algorithm must be implemented, and a route assigned to each flow. The processes of multiroute routing allow better use of the network resources, by distributing the traffic over a greater number of nodes. To achieve this, there must be a different metric, which allows multiple routes from the source node to the sinks, as in [16], where the Dijkstra algorithm is used with a metric composed of multiple parameters such as congestion level, signal level (RSSI), and the battery level of the nodes. This allows multiple routes to be generated to differentiate traffic according to QoS.

The SDN-WISE controller uses the Dijkstra algorithm to calculate the route at the lowest cost. By default, the metric it uses is the RSSI value. However, it is possible to use the topological knowledge offered by SDN to implement a metric that accumulates the amount of traffic scheduled for each node, without the need to send this information from the nodes. This is possible because the MAC layer will be TSCH and the traffic flows are fully defined in the traffic manager. The Dijkstra algorithm receives the topology that is defined by the nodes and each of their links. These links have assigned a weight, and this is the value that the algorithm uses to find the best route. However, using a metric for each link is not optimal in a WSN, because the nodes can only handle a unidirectional link in an instant of time. For this reason, a general metric is created over each node (*NodeUse*), which affects all its links. As the Dijkstra algorithm operates on the weights of the links, this value must be updated by adding the metrics of the nodes that make up the link (nodes *i* and *j*), see Equation (2). *NodeUse* is a traffic accumulation parameter. It is obtained according to the *Max_Deadline* in the flow table and the deadline obtained for each of the previously routed flows. In Equation (2), this is represented by a *deadline_ik_*, which is the deadline in the first node *(i)* of *link_ij_* for the flow *k*. The accumulation is on each of the previous routed flows, this means from *k =* 1 to *k = n*−1. The process on the second node (*j)* is equivalent with the flows *l* previously routed for this node.

(2) $$Weight\_link_{ij}=NodeUse_{i}\;+NodeUse{\;}_{j}=\sum_{k=1}^{n-1}\frac{Max\_Deadline}{deadline_{ik}}+\sum_{l=1}^{n-1}\frac{Max\_Deadline}{deadline_{jl}}$$

Therefore, the best route is defined by the sum of different weight links in each hop, Equation (3). Now the *link_ij_* changes in each hop *p*, so the nodes *i* and *j* depend on the hop *p*, represented as *i_p_* and *j_p_*. The process iterates over a list with *n* flows, in each iteration the route with less congestion is selected. Moreover, to ensure that resources are allocated for the highest priority flows first, the traffic manager organizes the flow list by priority and deadline.

(3) $$Weight\_flow=\sum_{p=1}^{\#hops}(\sum_{k=1}^{n-1}\frac{Max\_Deadline}{deadline_{i_{p}k}}+\sum_{l=1}^{n-1}\frac{Max\_Deadline\;}{deadline_{j_{p}l}}\;)$$

Another advantage of SDN in WSN is that, unlike the nodes, the controller has no processing limitations. Therefore, centralizing the processes allows the complexity of the algorithms to be increased or more intensive use to be made of them. In this case, the optimal routes are calculated, and the node and link metrics are updated each time the routing process assigns a route. Since the deadline determines the frequency with which traffic is sent, it is a factor that increases the metrics in each node.

In general, Algorithm 1 has the following stages: Obtain the highest deadline of the flows to be routed; take an entry of the traffic manager that are ordered by priority and deadline; use Dijkstra’s algorithm according to the topology and the parameters of the flow to calculate the route; and finally, use the deadline to update the metrics of the nodes used. Then repeat this process for each of the traffic manager entries. Therefore, the first routes to be calculated will have lower latency. Subsequent routes are distributed over the less congested nodes. This generates multiple routes to the sink, allowing transmissions and receptions not to be concentrated on the central nodes, balancing the load on as many nodes as possible and in turn increasing the *lifetime*.
**Algorithm 1:** Routing Accumulative Weight***G = (N, L);*** (Topology, **N is the set of nodes and L the set of Links**. Each Link is composed of Node1, Node2, and Weight);**TrafficDescriptor[n][4]; (Matrix with n flows and 4 parameters:** Priority, SourceNode, DestNode, Deadline);**MaxDeadline** = getMaxValue from TrafficDescriptor[n][4]**NodeUse[N];** (usage counter of each node)**for***i* = 1 → n **do** Path[i] = Dijkstra (G, SourceNode[i], DestNode[i]); **for**
*each Node in Path[i]*
**do**  NodeUse[N]+ = Maxdeadline/Deadline[i]; (accumulate the amount of traffic) **end** **for**
*each Link in G*
**do**  Link weight = NodeUse[Node1]+NodeUse[Node2]; (Update the weight of all Links) **end****end**

**Figure 3:**
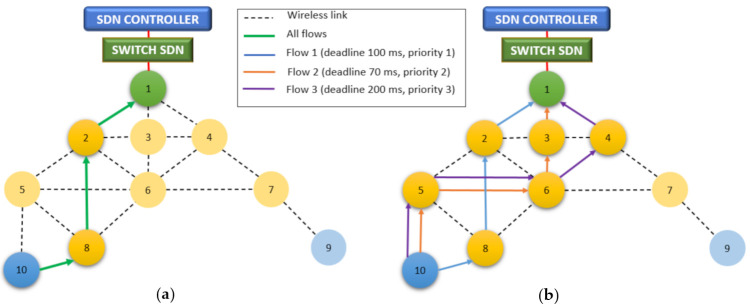
Flow between nodes 10 and 1. (**a**) Simple Dijkstra, based on Received Signal Strength Indicator (RSSI), (**b**) accumulated Dijkstra, balanced load.

Figure 3 shows a comparison between the Dijkstra algorithm based on RSSI, and the Dijkstra with the proposed metric. In the topology, node 10 has 3 flows organized according to their priority. If the deadlines are met precisely in 1 s, they will be satisfied: 10 packages of priority one, 15 of priority 2, and 5 of priority 3. Therefore, the load of node 10 will be 30 packages/s. As can be seen in Figure 3a, a route with three hops is defined where intermediate nodes 8 and 2 will have the full load of node 10 (30 packets/s). With the metric proposed in Figure 3b, there are two routes with four hops and one route with three hops. In this way, the traffic load for nodes 8 and 2 is reduced to 10 packets/s since the traffic has been balanced. The fact that the proposed metric has a higher number of total hops means that the network consumes more energy. However, the traffic is distributed over a larger number of nodes. This load balance allows a reduction of congestion and energy consumption in the central nodes. Furthermore, the use of different nodes increases the parallelism in the next stage (TSCH). In general, an increase in throughput and *lifetime* is achieved. Another advantage of using multiple paths is the increase in flexibility, as it is possible to use the alternate paths without waiting for the controller to reconfigure the network. This routing process is independent of the nodes, has no hardware limitations and can be modified directly in the controller, as the nodes only have to add the routes that are given as a result. This increases the flexibility of the network, as it is easily adaptable to environments requiring more specific metrics.

### 3.3. TSCH Scheduler in SDN-WISE

TSCH scheduler is the last process to be integrated. It is responsible for translating the routes calculated by the routing process so that transmissions are optimized by scheduling the receive and transmit slots for each node. The result obtained in this process will be the one that the controller sends to the nodes. The general problem is that most studies are focused on specific parts, omitting some important component for a TSCH SDN. Previous works [39,40] use the elements of the 6TisCH stack to optimize the TSCH Schedule in a centralized and hybrid manner. They also incorporate the use of tracks for high priority services. However, the tests are carried out with routes and cells installed manually in each node, which omits sending the scheduling to the motes and the signaling traffic this generates. Other works such as [36,41], address the need to associate SDN and TSCH in industrial applications. However, in this case, they are completely theoretical studies. Taking into account the need to treat the requirements of SD-WSN differently from classical SDNs, in [33], the authors propose combining TSCH with the SDN-WISE protocol in order to meet the mobility requirements of IWSNs. Since the results obtained are preliminary and do not address the feasibility of a real deployment, the same authors present in [17] the FTS-SDN protocol (Forwarding and TSCH Scheduling over SDN), which provides good results both in experiments and in simulation of real scenarios. However, the TSCH Scheduler and the control traffic that increases in an SDN over WSN deployments are not addressed in detail in this work either, since the study focuses more on the mobility of the nodes.

For all these reasons, a centralized TSCH Scheduler is implemented in this section, as part of a complete SDN system, which is completed in Section 3.4. The TSCH Scheduler receives the routes of the routing algorithm and the traffic manager to plan the data flows and meet the QoS requirements. In addition, the results of this scheduling will be sent to the nodes with the least possible signaling traffic. This process allows the scheduling of flows according to deadlines and reduces the delay according to their priority. To meet the deadlines, each flow is scheduled multiple times with a separation, which depends on the length of the slot frame and the deadline. Since the slot frame is cyclical in time, its length determines the longest deadline. Modifying the size of the slot frame according to the traffic manager deadlines allows these requirements to be met by minimizing transmissions, which reduces energy consumption. For each repetition of a flow within the slot frame, a channel rotation is scheduled. The delay is a parameter that was taken into account in the routing protocol and which in a TSCH network is related to the number of cells needed to reach the destination. Because of this, delay-sensitive flows are composed of a consecutive grouping of cells.

For the proposed schedule, Algorithm 2 is shown, where two matrices [NodesInRX] and [NodesInTX] are created with dimensions [NChannels][SlotframeLength]. One matrix is for the transmitting nodes and the other for the receiving nodes. These are in charge of storing the scheduled nodes. Every time a cell is going to be assigned, these matrices are accessed to verify that the node and the channel are not currently assigned.
**Algorithm 2:** TSCH Scheduler**Timeslot = 10 ms;****SlotframeLength;****NChannels;****TrafficManager[n][4];*(Matrix with n flows and 4 parameters: Priority, SourceNode, DestNode, Deadline);*****Path[n];** (The result of the Algorithm 1 for each entry in TrafficManager);**NodesInRX[NChannels][SlotframeLength];** (Matrix that store the scheduling in RX);**NodesInTX[NChannels][SlotframeLength];** (Matrix that store the scheduling in TX);**for***i* = 1 → n **do** Nodes[k] = getNodesfrom Path[i]; **for**
*j =* 1 → k **do**  Search Slot in NodesInTX for Node[j];  **if**
*NodesInTX[Slot] has Node[j] or Node[j+1]*
**then**   next Slot;  **end**  **if**
*NodesInRX[Slot] has Node[j] or Node[j+1]*
**then**   next Slot;  **else**   Search free ChannelOffset in this Slot and Schedule Tx and Rx nodes in NodesInRX, NodesInTX;   **if** last hop **then**    Schedule this Path with a deadline offset   **else**    Schedule Next hop;   **end**  **end** **end****end**

The process starts with the Path matrix generated in Algorithm 1, which has the routes for each of the flows. This was ordered by its priorities; the highest priority is the first one to be scheduled. The procedure to follow is decompose the route in hops; for each hop the availability of the nodes in that timeslot is checked, if any is scheduled it is passed to the next timeslot, where it is checked again, until one is found where both are available; in this timeslot a free channel is sought and added to the scheduling. When a route has been scheduled, the deadline is checked and the route is scheduled again, with the necessary timeslot shift.

In order to generate the scheduling, the following rules must be taken into account:

- The timeslots 1 and 2, configured as shared slots, only send control information of the SDN-WISE and TSCH.
- The nodes are half duplex and cannot transmit and receive at the same time. To avoid conflicts, a node can only belong to one matrix: [NodesInRX] or [NodesInTX] in the same timeslot (Conflict-free).
- To avoid interference between the nodes, this algorithm does not reuse the channel in the same timeslot. Each parallel transmission has a different channel (Search free ChannelOffset).
- Conflict-free nodes can be scheduled in parallel.
- Scheduling the traffic manager deadlines for each flow, with a repeated path using a deadline offset.
- The deadlines must be met within the slot frame and when linking to the following one.
- Flows that are repeated in the slot frame must be scheduled with a channel rotation.

The following are the general considerations to be taken into account when deciding on the size of the slot frame:

- Bandwidth and energy consumption. The slot frame is directly related to energy consumption. Short slot frames will increase the bandwidth and energy consumption, while long slot frames will reduce the energy consumption and bandwidth.
- Time to update the topology. The controller is usually updated at the beginning of each slot frame in the shared slots, used by the nodes to flood the network with beacons, which allow the construction of the topology. Therefore, in stable networks, the slot frame can be extended. In specific cases, it is possible to add shared slots in different points of the slot frame.
- To ensure that communication takes place on all available channels, the number of slots in the slot frame must be a prime number.

The use of different deadlines for each flow, makes the advantages of the long and short slot frames available, since it is possible to have greater bandwidth and short deadlines in a specific flow, without needing to repeat the other flows of the slot frame, thus reducing the energy consumption.

**Figure 4:**
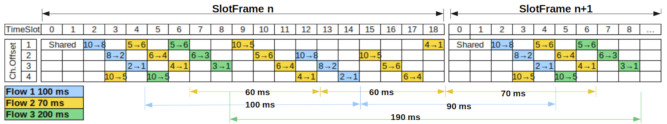
Scheduling flows with different deadlines.

Figure 4 shows the scheduling of the three flows represented in Figure 3b, in a slot frame with 19 timeslots (190 ms). This length allows the longest deadline (200 ms) to be met for each repetition of the slot frame. The other deadlines are scheduled multiple times with a rotation of the channel offset, so that the time between the flows is always less than the deadline. If this schedule were cut to nine timeslots, which is the minimum size to contain the three flows, these would have a periodicity of 90 ms, which is close to meeting all deadlines. However, meeting deadlines with too much slack considerably increases energy consumption. This is why it is important to plan flows dynamically.

### 3.4. Modification SDN WISE

The main contribution of this paper is a TSCH network fully defined by software, integrating multiple processes from the application plane. These processes allow a slicing by flows to apply different quality of service parameters. To achieve this, the previous sections have explained the logical and physical segmentation of the routes to be assigned to each flow. In this section, the operation of the nodes is explained. For this purpose, significant modifications have been made to SDN-WISE. Firstly, the MAC has been changed, and secondly, the operation of the OpenPath package has been extended to carry out the TSCH scheduler.

Modifications were made to version 3.0.6 of the SDN WISE Contiki, which is available in an open format. Because Contiki has a new version with improved timers for TSCH and support for more modern nodes, the project was moved to this platform. In order to test this SDN deployment in the WSN, the architecture is as proposed in Figure 1. OpenMotes B will be used for the Sink and Nodes.

The following explains the modifications made to the OpenPath package, which will be called OpenPathTSCH, to encode the TSCH Scheduler information and shows a detailed example for validation as well as showing how the nodes carry out the decoding of the received frame.

**Figure 5:**
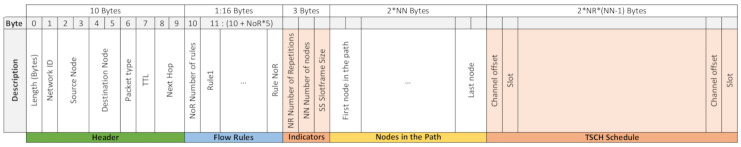
Structure of the OpenPathTSCH packet. Generic proposal.

Modification of the OpenPath packet. To include TSCH functionality without increasing control traffic, the OpenPath package is extended by adding specific fields for the assignment of TSCH Scheduler, see Figure 5. The added fields have been colored “orange”. There are two groups of bytes named “Indicators” and “TSCH Schedule”. The group “Indicators” is made up of 3 bytes coming after the Flow Rules.

- NR (Number of Repetitions, 1 Byte): This is a field that indicates how often the route is repeated within the slot frame. It is directly associated to the deadline of the traffic flow that will use this route and to the size of the slot frame. It is used to inform the node of the amount of Tx cells that it must install. In addition, the MSB (Most Significant Bit) indicates the direction of flow, so a 0 is used for the flow from the Sink to the nodes (Downlink) and a 1 for the traffic from the nodes to the Sink (Uplink).
- NN (Number of Nodes, 1 Byte): The total number of nodes that make up the route. In the original OpenPath this parameter was not necessary since it was possible to start from the packet length and the number of Flow Rules. In the case of the deployment presented in the paper, it is necessary give the information so that the scheduling bytes of each node are correctly delimited.
- SS (Slotframe Size, 1Byte): Is a parameter that has implications for throughput and energy efficiency, since for the same cell allocation, increasing slot frame size means more cells with the radio off, and reducing it means more frequency between transmissions. However, the nodes must have the same configuration so this is explicitly done with this byte. This modification of the SS significantly increases flexibility. As shown in the previous section, the adequate selection of the size allows the deadlines to be tightly scheduled, thus increasing energy efficiency.

The bytes associated with the “TSCH Scheduler” have been placed at the end of the packet. Each cell in the schedule is encoded with 2 bytes, one for the Channel Offset and one for the timeslot. The number of cells added here depends on the NN and the NR. Each node must have one cell per route repetition, therefore the number of cells of a node will be equal to the NR. These cells are ordered in the same order as the route, that is, the first NR cells correspond to the first node in the route and so on.

**Figure 6:**
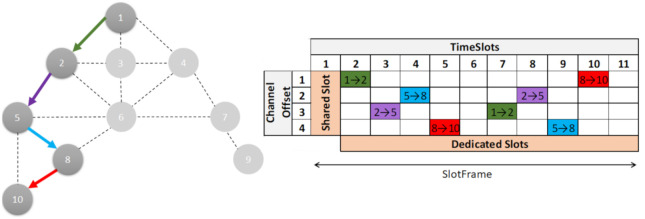
Topology of 10 nodes. Time Slotted Channel Hopping (TSCH) Scheduler of a 5-node Path with NR = 2.

**Figure 7:**
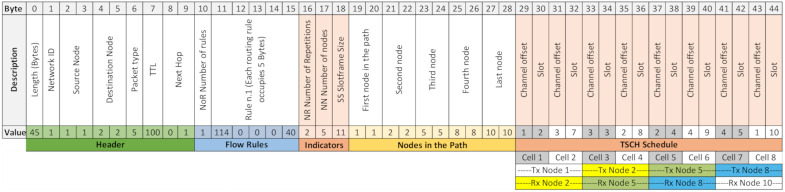
OpenPathTSCH packet structure for a 5-node route.

Validation of the use of OpenPathTSCH. To validate the mechanisms of the OpenPathTSCH packet, a topology with 10 nodes was used. In this example, the Path is formed by 5 of them as shown in Figure 6. Downlink traffic is used, where the MSB of NR is at 0. On the selected path, the traffic must go from node 1 to 10 and hop at each of the intermediate nodes. It will be verified that from the OpenPathTSCH packet the behavior of the MAC TSCH layer in all the nodes of the route in this example (1-2-5-8-10) can be modified. This flow will have 2 NR within the slot frame, which has a length of 11 timeslots, as shown in Figure 6. This image only shows the scheduling of the nodes belonging to the Path. The timeslots in the slot frame must be assigned in the order of the traffic flow to prevent packets from accumulating in the buffer of the nodes. In this example, therefore, node 1 is the first assigned and must be transmitted to node 2 in Channel Offset 1, timeslot 2 and also in Channel Offset 3, timeslot 7 according to the TSCH scheduler. This is encoded in the OpenPathTSCH package in the bytes dedicated to the TSCH scheduler using 4 bytes as follows (Channel Offset-Slot-Channel Offset-Slot): 1-2-3-7. As it is the source node there are no reception cells for this flow since the schedule only contemplates the transmissions since the receptions are implicit. The transmission information coded in the Cells can be grouped as the transmission information of each node. The length of each grouping depends on the Number of Repetitions. Figure 7 shows the OpenPathTSCH packet for the specific case of the 5-node route. In this image, the byte group that includes the two Cells with information of Node1 is labelled as “Tx Node1”. The group occupies 4 bytes because NR = 2 in this case. The grouping of the Cells is organized according to the order of forwarding, that is, first the group of Cells of the first hop in the route and then the group of Cells of the next hop, and so on.

Similarly, node 2 must transmit to node 5 in Channel Offset 3, Slot 3 and in Channel Offset 2, Slot 8, this will be encoded again using 4 Bytes 3-3-2-8. This node must have reception cells to receive the packets sent by node 1, in cells 1 and 2: Channel Offset 1 Slot 2 and Channel Offset 3 Slot 7. As these correspond to the transmission bytes of node 1, they are not added to the packet. Thus, the scheduler coding will be composed only of the transmission information of each node and the reception cells are deduced from the transmission cells of the previous node. The scheduler of the rest of the nodes would be, Node 5: 2-4-4-9 and for Node 8: 4-5-1-10. For Node 10 there would not be codification of the scheduler, since only the transmission is codified, and this node only receives.

Decoding the elements in the frame. The receiving node must decode the information contained within the received frame, starting with the header that has a fixed structure of 10 Bytes where it first compares the values of the destination field and Next hop, to verify that this frame belongs to it. The following structure is variable and is analyzed based on the type of packet, in this case, OpenPath is type 5. Therefore, the next byte determines the length of the Flow Rule, which for this example is 6 bytes because it has a single matching rule. After the rules, the Number of Repetitions, NR = 2, would be indicated for the example in Byte#16. The value of NR also indicates the direction of flow and values lower than 128 are downlink (MSB = 0). The difference between uplink and downlink is that the assignment of the transmitting and receiving nodes is done in reverse, as the OpenPathTSCH packet is transmitted in the opposite direction to the flow. Below, the Number of nodes is indicated, in this case NN = 5, since there are only five nodes belonging to the route, even though there are 10 in the WSN. Next, the length of the Slot frame is indicated, LS = 11, as shown in the TSCH Scheduler of Figure 6. Then, each of the nodes on the Path is compared until its ID is found, which will give its position within the Path. If the node that has received the packet is 5, the value it gets as position will be equal to 3, bytes 23 and 24 of Figure 7. It then knows which is the destination of the route that corresponds to the last node, in this case, node 10, which is the node before it in the route. In the case of the example, it would be node 2, and the next node where it must forward the packet, which for the example corresponds to node 8.

Finally, there are the scheduling bytes, where the specific scheduling for the position on the Path must be obtained. The process is as follows: The number of cells that must be read is equal to the NR. The first cell that corresponds to it depends on its position on the Path, 1 + (position−1) × NR. For this example, node 5, which is in position 3, must read cells 5 and 6 or, in other words, bytes 37 to 40 of Figure 7. In general, the initial byte of the scheduling will be variable. As we have seen, its position depends on the amount of flow rules and nodes in the route. It can be expressed as follows, Byte#(10 + NoR × 5 + 3 + NN × 2 + 1). The length of this scheduling will be determined according to Equation (4) by the NN, the NR, and the two bytes needed for the channel and slot.

(4) $$L=2\cdot NR\cdot \left(NN-1\right)=2\cdot 2\cdot \left(5-1\right)=16\;bytes$$

The maximum size of 802.15.4e packets is 127 bytes. Taking away the 11 bytes required by the MAC layer results in a maximum SDN-WISE TSCH packet size of 116 bytes. For OpenPathTSCH this is equivalent to Equation (5), where *L* is the value obtained by Equation (4).

(5) Header+1+NoR·5+Indicators+2·NN+L<=116 → 45 <=116

For scheduling with a typical slot frame length equal to 1 s (101 Slots) and a flow with 4 hops, the maximum number of repetitions that could be scheduled for this flow would be 11, which is approximately equivalent to 1 packet every 100 ms.

## 4. Performance Evaluation

### Scenario and Metrics

For the tests, the scenario shown in Figure 3 was used; a sensor network made up of 10 nodes and the controller that receives the WSN information from the Sink, node 1. Nodes 9 and 10, in blue, will generate traffic with different levels of priority. The rest of the nodes (the yellow ones) are in charge of routing the traffic generated. The network has three types of traffic, with different priorities according to the delay and packet loss rate, as indicated in Table 1. Each type of traffic is assigned by the controller to a different route determined by the expected congestion at each node. For each of the routes, a TSCH schedule is generated to suit the requirements. Table 2 shows the values of the parameters used in the simulation. Generally, the default values were used except for MAC retransmissions, which by default have a value of 7 and the burst, which are enabled by default.

The following is a description of the three tests that were carried out together with the metrics to be evaluated, both through simulation and in the testbed.

Test 1. Protocol Efficiency

Firstly, the general characteristics of the deployment are evaluated according to the following metrics:

- Convergence time: The time needed for the controller to create the topology image based on the report packages it receives from each of the nodes.
- Number and size of control packets: The exchange of beacons and report packets is essential traffic for the operation, but it must be controlled and must not affect the configured flows.
- Configuration time: The time that elapses from when the controller sends an OpenPathTSCH and in the destination node the transmission begins.

For tests 2 and 3, five data traffic flows have been defined, as shown in Table 3, that will be sent during 42,000 timeslots (7 min). The flows are defined in the scheduler by a priority, a deadline, the source node, and the destination. This allows the scheduler to allocate the necessary resources to provide optimal quality of service. The delay and packet loss determine the priority level (first column of Table 3), and are parameters that must be kept in the implementation for each of the flow tests.

Test 2. Delay in the Configuration

In this case, the performance of the deployment is obtained. The metrics that will be evaluated are:

- End to End Delay: Number of timeslots needed to transmit the source packets to the destination.
- Packet inter-arrival time: The number of timeslots that elapse between each reception of the same flow in the destination node. This is the main metric, as it allows for the evaluation of how the network adapts to the strict time requirements. From this metric, the satisfaction rate of the service quality requirements is obtained, which is included in the Deadline Satisfaction Ratio (DSR).
- Power consumption: In a TSCH network, this can be obtained from the time the node has spent in the transmission and reception states. The size of the packets affects this time. The ratio of the node’s receive-and-transmit time to the total time is the RDC (Radio Duty Cycle).
- Network lifetime: Is the total time of the operating network until the first node is downloaded. To calculate this, we take into account the values in Table 4 (obtained from the OpenMote datasheet), and the time each node spends in these states.

Test 3. Multiple Transmitters

In these tests, nodes 9 and 10 will transmit packets of different priorities according to Table 3. During transmission, the failure of node 8 will be simulated by turning it off 5 min after the first packet is sent. This will allow the change in the Delay and packet loss for each priority to be measured. Packets from flow 1 must be re-routed through flow 2′s route without controller intervention. In addition, the configuration time must be measured. In this case, the measurements of Delay and packet loss of node 10 are divided into three instants: Initial (all nodes are operational), Fault (Node 8, off), and with the new routing schedule.

## 5. Results

### 5.1. Simulation

Test 1. Protocol Efficiency

**Figure 8:**
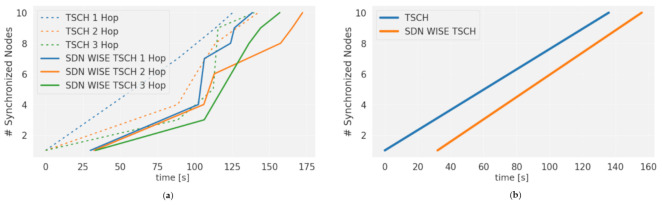
Software Defined Networking solution for Wireless Sensor Networks (SDN-WISE) TSCH convergence time with TSCH synchronization; (**a**) Convergence time; (**b**) Trend Convergence time

The network Convergence time depends mainly on the TSCH synchronization. Figure 8a shows the result for a 1-s scan time on the four channels with one, two, and three hop networks. As can be seen, SDN convergence has a delay (continuous lines), because Topology Discovery starts after TSCH synchronization for each node. The increase in this time is not significant. According to Figure 8b, the network takes 32 s more to be ready to send the first package.

The total Convergence time for SDN-WISE TSCH is given by TSCH’s Average Synchronization Time (*T_S-TSCH_*) and Topology Discovery Time (*T_TD_*) according to Equation (6)

(6) $$T≅T_{S-TSCH}+T_{TD}=T_{S}\cdot NH+T_{TD}$$

The TSCH’s Average Synchronization Time is determined by Equation (7) according to [42] multiplied by the number of hops (NH).

(7) $$T_{S}=M_{T}\cdot \frac{\left(C+1\right)}{2\cdot PDR}=M_{T}\cdot 2.5$$

*M_T_* is the Average period for EB transmission, in our case 16 s. Taking into account that *C* is the number of channels in TSCH schedule, 4 in the schedule used and the PDR is 1, by applying these values to Equation (7) a TS of 40 s is obtained. Considering that each node sends the information to the controller by the shortest route and taking into account the worst case, the most distant nodes, three hop routes would be used for the proposed topology, where *NH* = 3. Thus, the total time for synchronization is equal to *TS-NH* = 120 s, similar to that obtained in other works, see Figure 13 of [43], where the Average Synchronization Time for 3 hops is around 155 s.

In addition, according to Equation (6), *T_TD_* must be considered, which in our case is around 32 s. With this, we finish with *T* ≈ *T_S_* · *NH + T_TD_* ≈ 120 s + 32 s ≈ 152 s approximately, which are the 152 s average obtained in the simulations.

**Figure 9:**
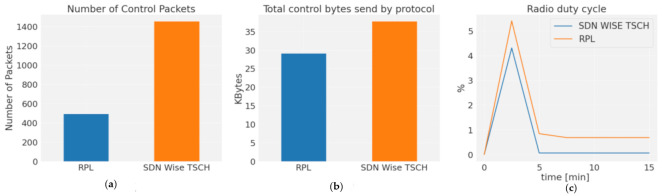
Control traffic generated by Routing Protocol for Low-Power and Lossy Networks (RPL) vs. SDN-WISE.

After the convergence phase, it was observed that SDN-WISE continues to exchange messages and inform the controller of the topology on a regular basis. However, those protocols that make use of the RPL protocol are stabilized and exchange a smaller number of control packets [12]. The number of packets generated by SDN-WISE is significantly higher than that of RPL, Figure 9a. However, part of this difference is compensated by the byte size of the RPL packets, which is twice as large as the total number of bytes sent by each protocol, as shown in Figure 9b. Although the number of bytes sent is still lower for the distributed protocol, most of these bytes are transmitted in the initial phase, while in SDN it is done constantly, so the initial peak in the RDC caused by TSCH synchronization will be increased; see Figure 9c. The stability phase is still lower for SDN-WISE TSCH, because the controller has not configured any slots, while in distributed protocols, a certain number of slots per neighbor is automatically enabled, although it is not necessary.

**Figure 10:**
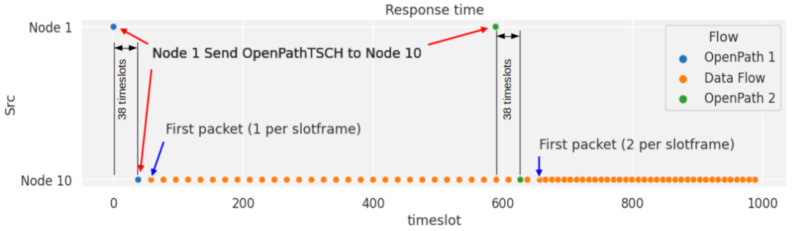
Response and change of scheduling time.

The stability phase is still lower for SDN-WISE TSCH, because the controller is not set to any slot. The responsiveness of the controller to change the behavior of the WSN is the Configuration time. This is composed of two elements, the node arrival time and the scheduling processing and installation time, which is in charge of synchronizing packet generation. This last element is totally dependent on scheduling, while in distributed protocols, a certain number of slots per neighbor is automatically enabled, even though this is not necessary. Figure 10 shows the sending of two OpenPathTSCH, one at timelot 0 and the other at timelot 590. For the first configuration packet, the node configures the sending frequency to 1 packet/slotframe. For the second packet, the previous schedule is changed and the sending frequency is changed to 2 packet/slotframe. Because OpenPathTSCH is transmitted on Shared Slot, the arrival time at the node is equivalent to the Shared End to End Delay (Shared_E2E). This time depends on the slotframe size, the number of hops to the destination node (NH), the number of shared slots, and the PDR. For a PDR of 100% according to Equation (8), we would have 38 timeslots. The new schedule is executed from the second active timeslot, since the first one is used to synchronize packet generation.

(8) $$Shared\_E2E=\frac{\left(NH-1\right)\cdot SlotFrame\_size}{Number\_SharedSlot\cdot PDR}\;$$

Test 2. Performance Evaluation

To evaluate the performance of SDN-WISE TSCH, a transmission was made over 42,000 timeslots for each of the flows of node 10. It was made with each one separately and the test was repeated with all the flows transmitting. In this way, it was possible to evaluate how the protocol adapts to the configured transmission frequencies without interfering with the other flows. Node 10 flows with 100 ms, 70 ms, and 200 ms deadlines were used. The scheduling algorithm used by SDN-WISE TSCH allows multiple transmission slots to be assigned to suit the needs of each flow. As obtained in the scheduling section, the optimal slot frame size for this case is 19 slots, where a total of 6 transmission slots are assigned to node 10. The slot frame size and number of transmission slots are configured so that the Packet inter-arrival time is always within the deadlines.

**Figure 11:**
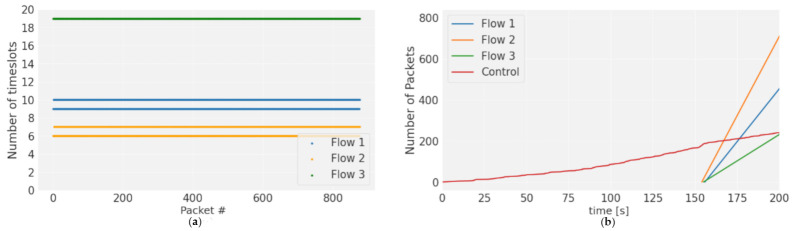
(**a**) Packet inter-arrival time for all flows, (**b**) Number of packets by flow.

The result is shown in Figure 11a, where the behavior is the same in the isolated and combined case. The flows behave in a totally deterministic way, according to the scheduling shown in Figure 4. Flow 1 has two repetitions assigned and they are separated with 10 slots within the same slot frame and with 9 slots between slot frames. For this reason, two lines of the Packet inter-arrival time are shown. The same behavior shows flow 2 with 6 and 7 slots, and only flow 3 has a single value as it coincides with the size of the slot frame. Figure 11b shows the different flows in the network, and the data flows have a completely linear growth, as they do not show any interruption. This is due to the fact that packet generation is synchronized with the slot frame, so each of the flows has the necessary resources assigned, avoiding losses and guaranteeing 100% DSR. For the case of the control flow, the initial part has a slow growth, since only the EB are exchanged. As the nodes are synchronized, there is a more noticeable increase in the flow, due to the introduction of the SDN-WISE TSCH control packages.

SDN-WISE TSCH maintains the transmission and reception characteristics in each of the flows regardless of whether they are isolated or combined. This is because, with the routes, TSCH scheduling, and packet marking, a slicing has been created that allows network resources to be allocated and conserved for each of the flows. In this way, there is no interference under normal conditions.

As has been observed so far, in this deployment of SDN-WISE TSCH, all information flows can be guaranteed so that neither excess control traffic nor other flows alter the scheduling of the network, thus obtaining high performance of the information flow.

**Figure 12:**
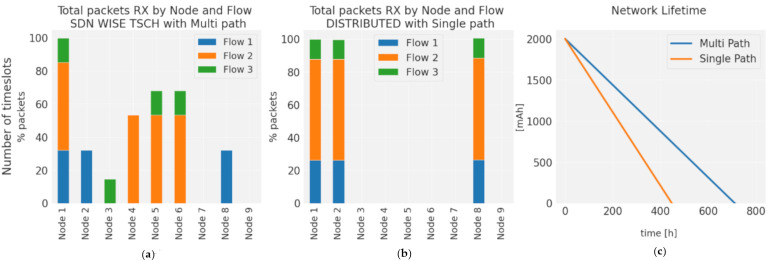
Distribution of flows over the nodes; (**a**) SDN WISE TSCH; (**b**) single path for all nodes; (**c**) network lifetime for both.

Figure 12a shows the advantages of using multiple paths. SDN-WISE TSCH differentiates flows by priority, which allows multiple paths to be assigned, distributing traffic over a larger number of nodes. Figure 12b shows the use of the same path for all flows. This distribution has a direct impact on network performance and Network lifetime, as shown in Figure 12c. The use of a single path generates a premature depletion of the central nodes. For the case of the single path, node 2 has the highest consumption and is 33% higher than the consumption of node 6, which is the highest consumption for the case of multipath. Although the general consumption in the case of multipath routes is 9% higher due to the greater number of hops, this consumption is not centered on a single node, it is distributed over a greater number of nodes, which allows the Network lifetime to be increased.

**Figure 13:**
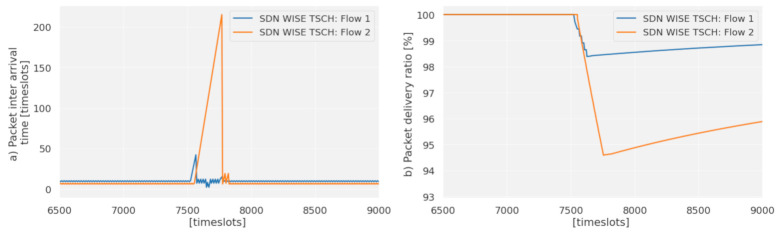
Path change after node 8 failure; (**a**) Packet inter arrival time; (**b**) Packet delivery ratio

One of the advantages of distributed protocols is the ability to reconfigure autonomously, which allows the network to adapt to failures quickly. In a traditional SDN, this would require that the nodes instruct the controller to perform a reconfiguration. This would lead to a delay while the topology is recalculated, and the configuration is sent to the nodes. These delays and the excess of control traffic that occurs limit the use of centralized approaches, and for this reason in SDN WSN some decisions are left to the nodes. A system of states allows the nodes to alter the decisions of the controller based on local information. In this case, this possibility and the use of multiple routes is exploited to preserve the PDR of priority flows, redirecting the highest priority traffic when there is a failure. Figure 13a shows the Packet inter-arrival time for node 10 during 7000 timeslots is kept constant while fulfilling the 100 and 70 ms deadline for flows 1 and 2. At 7500 timeslots, node 8 fails, and there is an increase in the Packet inter-arrival time of flow 1 of up to 40 timeslots. This is the time it takes for the buffer to exceed the configured occupancy limit, in this case, three packets. With the current generation period of flow 1 (10 timeslots), the limit is reached after 30 timeslots. The packet generated after reaching the limit is routed through the path assigned to flow 2. At this moment, the PDR is stabilized for flow 1 Figure 12b, in exchange for losing packets of lower priority from flow 2.

**Figure 14:**
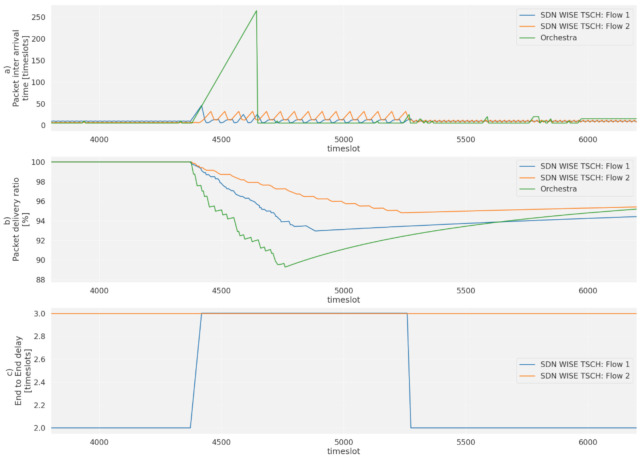
Comparison between SDN WISE TSCH and Distributed Scheduling.

The peak that occurs in flow 2 is due to the controller reconfiguration time. After these 200 timeslots (2 s) the controller has sent a new schedule that meets the requirements set for each flow. The use of the state system, besides reducing the loss of packets by changing paths, allows the controller to be informed about the failure of node 8 immediately, thus skipping the time it takes the topology discovery to discover the failure, shortening the reconfiguration time to comparable levels of distributed protocols, as shown in Figure 14 where a comparison has been made with Orchestra. When a failure occurs, in timeslot 4400, the packet inter arrival time increases to 250 timeslots for the distributed protocol as can be seen in Figure 14a. However, for SDN-WISE it goes up only to 40 timeslots, while balancing the traffic of flow 1 and 2 on the second route. Thus, the packet loss is mitigated by the use of this route; see Figure 14b. However, for the distributed protocol, it falls until the reconfiguration time is reached. For the case of SDN-WISE this time can be seen in the change of the End-to-End Delay of Flow 1; see Figure 14c. When there is a failure the flow passes through the path of flow 2, so its End-to-End Delay is increased to the level defined for route 2, in this case, three timeslots. When the controller reconfigures the network, the flow returns to the End-to-End Delay of path with priority 1.

Test 3. Multiple Transmitters

The last parameter to be evaluated is the behavior of the network with additional traffic from other nodes. In this case, two flows from node 9.

**Figure 15:**
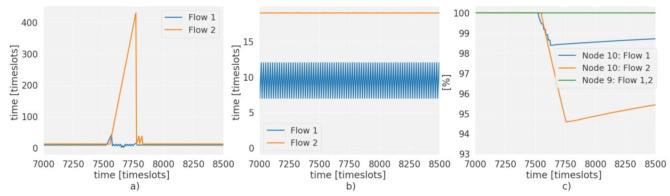
Effect of multiple transmission nodes in test topology; (**a**) Packet inter arrival time (node 10); (**b**) Packet inter arrival time (node 9); (**c**) Packet delivery ratio

Figure 15a shows the behavior of node 10, with a constant behavior up to 7600 timeslots, where the failure of node 8 occurs. Flow 1 is forwarded on path 2 and there is a packet loss (Figure 15c), for flow 1 until it changes path and for flow 2 until the controller reconfigures. All this process generates additional control traffic that must be managed so that it does not affect other flows. In the case of node 9, the Packet Inter-arrival time for flow 1 of node 9 ranges from 7 to 12 timeslots (see Figure 15b) because the algorithm has assigned two flows, one in slot 3 and another in slot 15. This configuration allows the deadline requirements to be met, and shows a cyclic behavior during the whole test, even in the moments when node 8 fails and node 10 requests a reconfiguration that increases the control traffic. No changes or alterations are seen in any of the flows. After these tests, it is verified that under normal conditions and alterations external to the node, a slice can be guaranteed for each flow and there is no interference between them, unless a network failure occurs, in which case the affected node will modify the packet sending to minimize the impact of the failure on the higher priority flow. The parameters measured in Figure 15b show how there is no alteration in the flows of node 9, the PDR is maintained at 100%, and there is no unforeseen fluctuation in the Packet Inter-arrival Time.

In the tests shown in Figure 15, flow 2 of node 9 was assigned a slot frame repeat, representing a Packet inter-arrival time of 190 ms. However, the deadline required for this flow is 300 ms, and meeting it by such a wide margin implies an excess of transmissions that can be avoided. For this reason, the scheduling algorithm chooses the size of the slot frame according to the flow with the highest deadline configured in the traffic descriptor.

### 5.2. Testbed

To check the deployment in a more realistic way, 10 Openmote B was used. The transmission power of the nodes was limited to −15 dB to achieve the multihop network that corresponds to Figure 3. In addition, the number of channels was reduced to 2 to reduce the number of measurement nodes (Sniffers). This channel reduction does not represent a performance loss for the tests performed. The Sink is composed of a combination of an Openmote B and an ODROID N2, which establishes the connection to the controller via Ethernet.

**Figure 16:**
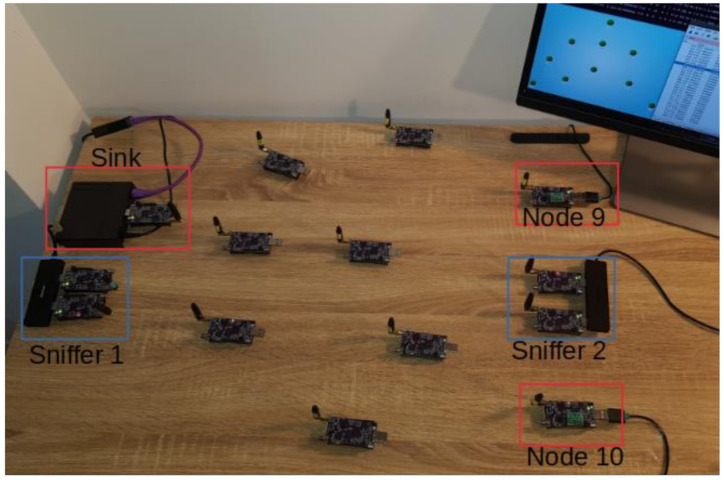
Testbed for 10 nodes topology.

Figure 16 shows the real elements that compose the network, 10 nodes including the sink, and 4 additional OpenMote B configured as sniffers that allow the traffic from nodes 9, 10, and the Sink to be captured. The captured traffic is used to measure the performance obtained. Since they cannot be synchronized with the TSCH, they capture the traffic on a single channel. For this reason, the number of channels has been reduced and two-node sniffers have been created, which allow all scheduled traffic to be captured.

**Figure 17:**
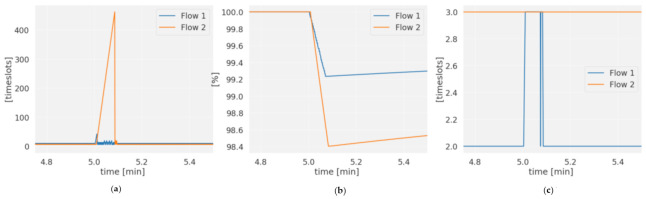
Performance with simulated failure in testbed; (**a**) Packet inter arrival time; (**b**) Packet delivery ratio; (**c**) End to End Delay

The tests carried out show behavior consistent with the simulation section, where determinism is maintained on a stable network. Figure 17 shows how the flows maintain stable behavior up to 5 min, where the node 8 has configured to turn off the radio. In this instant, a peak is generated in the Packet inter-arrival time of the flow 1 of 40 timeslots (see Figure 17a) exactly as in the simulation, since it is a value that depends only on the local occupation of the buffer. When these 40 timeslots are reached, the node exchanges the paths of the flows, which transmits the packet loss to flow 2 and attenuates those of flow 1, obtaining the results of the PDR shown in Figure 17b.

As long as the paths are exchanged, the End-to-End Delay of flow 1 will be the one with route 2, in this case three timeslots; see Figure 17c. This behavior extends until the controller resets the paths with a new schedule. This Reconfiguration time corresponds to the peak of the Packet inter-arrival time of flow 2, 480 timeslots. This represents a 20% increase over the maximum value obtained in the simulation. This increase is due to the use of shared slots, where there are collisions that are increased in the real topology. As in test 3 of the simulations, none of the metrics of node 9 is affected by the flows of node 10 or the failure of node 8.

## 6. Conclusions and Future Work

This approach allows the optimization of TSCH scheduling in time and frequency, while guaranteeing 100% compliance with deadlines in stable conditions. The use of multiple routes and priorities increases the availability of priority flows and increases Network Lifetime by distributing consumption over multiple nodes. The implementation of different states decreases the limitations of centralized protocols, such as the response time of the controller, which is reduced by reporting failures at the time they occur, eliminating the delay of the discovery protocol. Although there is a greater amount of control traffic, this can be limited by the number of shared slots in the scheduling.

The scheduling, marking, and resource allocation from the controller allows each node to differentiate the resources belonging to each flow, achieving a complete slicing between all traffic circulating on the IWSN. The use of a single configuration packet for each flow, such as OpenPathTSCH, results in a negligible overhead for the network, while increasing flexibility, by being able to assign and remove cells from the schedule without affecting other traffic. Finally, the centralization and combination of the previous elements allows a highly deterministic network to be obtained, where it is possible to guarantee the quality-of-service metrics that are configured for each flow.

As a future work, we will work with the same architecture without generating additional load, through an in-band network telemetry system. This will allow the implementation of more complex systems for the operation of the nodes, such as blocking physical channels or time slots with higher traffic. Support for mobile nodes can benefit from centralized scheduling, since instead of the topology adapting to change, multicast groups can be generated, which will communicate with the mobile node in the same timeslot. Since not all tasks are continuous, it is possible to increase dynamism and optimize the use of network resources with the implementation of a system of temporary resource allocation, requested directly by each node, and which will be required only when some local condition is met.

## Figures and Tables

**Table 1:** Priority classification.

Priority	Time Delay	Packet Loss Rate	Data Type
1	Sensitive	Sensitive	Events
2	Non-sensitive	Sensitive	Images and periodic data
3	Non-sensitive	Non-sensitive	Telemetry data

**Table 2:** TSCH parameters used in the simulation.

Simulation Parameters	SDN-WISE TSCH
Simulation time	7 min (42,000 timeslots)
Number of channels	4 channels
Join sequence	4 channels
EB period	16 s
MAC retransmissions	0
Burst	Disabled
Keep Alive Period	60 s
Timeslot	10 ms

**Table 3:** Data traffic flows.

Priority	Deadline	Flow
1	100 ms	10 🡢 1
1	150 ms	9 🡢 1
2	70 ms	10 🡢 1
2	300 ms	9 🡢 1
3	200 ms	10 🡢 1

**Table 4:** Operation modes and Power consumption for OpenMote B.

Mode of Operation	Consumption
Microcontroller–Mode: Sleep	1.3 µA
Microcontroller–Modo: Active	7 mA
Radio–Rx	20 mA
Radio–Tx	24 mA
Radio–Sleep	1 µA

## Data Availability

Not applicable.
